# (*E*)-3-Thia-1,5(1,3)-dibenzena­cyclo­undeca­phan-8-ene-6,11-dione 3,3-dioxide

**DOI:** 10.1107/S2414314620014649

**Published:** 2020-11-24

**Authors:** Sambasivarao Kotha, Naveen Kumar Gupta, Saima Ansari

**Affiliations:** a Department of Chemistry, Indian Institute of Technology Bombay, Powai, Mumbai - 400076, India; Goethe-Universität Frankfurt, Germany

**Keywords:** macrocycles, Grignard reaction, ring-closing metathesis, thia­cyclo­phanes, crystal structure

## Abstract

The molecular structure of the title cyclophane, C_20_H_18_O_4_S, has two benzyl groups, a sulfone group, and two carbonyl groups adjacent to a double bond. The phenyl rings do not show intra­molecular stacking.

## Abstract 2

The crystal structure of a sulfone-containing meta­cyclo­phane is discussed.

## Graphical abstract

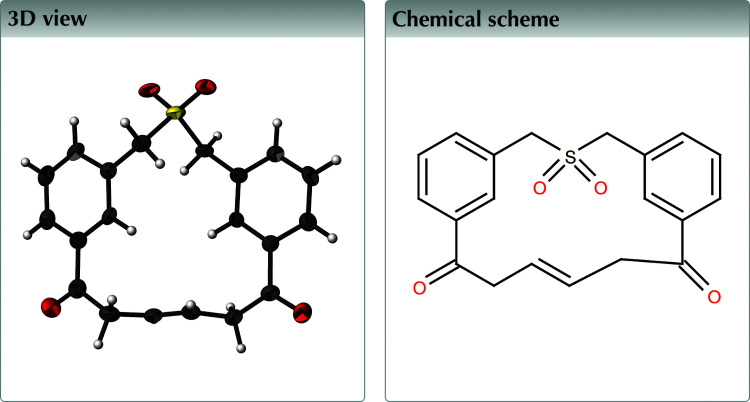

## Structure description

Cyclo­phanes (Cram & Helgeson, 1966 ▸; Kotha *et al.*, 2015 ▸) have become useful targets because of their unique structural features (Knobler *et al.*, 1986 ▸). Their shapes are the main cause for their applications in supra­molecular chemistry (Xu *et al.*, 2008 ▸), material science (Yu *et al.*, 2006 ▸) and medicinal chemistry (Lee *et al.*, 2002 ▸). To this end, the synthesis of sulfur-containing cyclo­phanes (thia­cyclo­phanes) has become of great inter­est for chemists (Nicolaou *et al.*, 2010 ▸).

**Figure 1:**
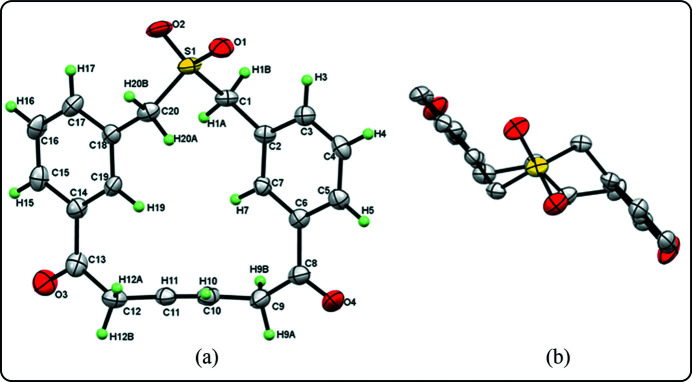
The mol­ecular structure of the title compound **1**, (*a*) showing the atom-numbering scheme and (*b*) top view (H-atoms are omitted for clarity). Displacement ellipsoids are drawn at the 50% probability level.

We have prepared novel thia­cyclo­phanes using a simple strategy involving the Grignard reaction and ring-closing metathesis as key steps (Kotha *et al.*, 2020 ▸). In this work, we present the single-crystal XRD study of the thia­meta­cyclo­phane **1**, which has two benzyl rings attached to an SO_2_ moiety and a bridge at the *meta* positions of the phenyl rings containing two carbonyl functions connected by a double bond (Fig. 1*a* ▸). The angles between the S atom, the *ansa*-bridging methyl­ene C atom and pivot atom of the phenyl ring *i.e.* S1—C1—C2 [114.1 (3)°] and S1—C20—C18 [114.8 (3)°] are slightly widened compared with an *sp* ^3^-hybridized carbon atom. The structure has completely out-of-plane phenyl groups with no intra­molecular inter­action between them, as is evident from the top view of the compound (Mitchell & Lai, 1984 ▸) (Fig. 1 ▸ *b*).

**Figure 2:**
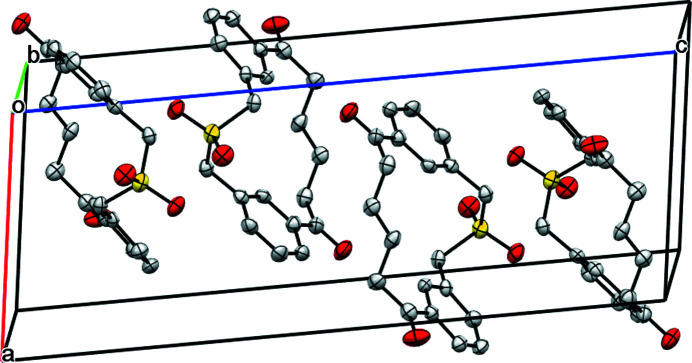
Crystal packing of the title compound viewed along the *b* axis. H atoms are omitted for clarity.

With respect to inter­molecular inter­actions, no π–π stacking between adjacent phenyl rings is observed, as is evident from the packing diagram shown in Fig. 2 ▸. The phenyl rings do not share any overlap. Inter­molecular hydrogen bonding is also absent.

## Synthesis and crystallization

**Figure 3:**
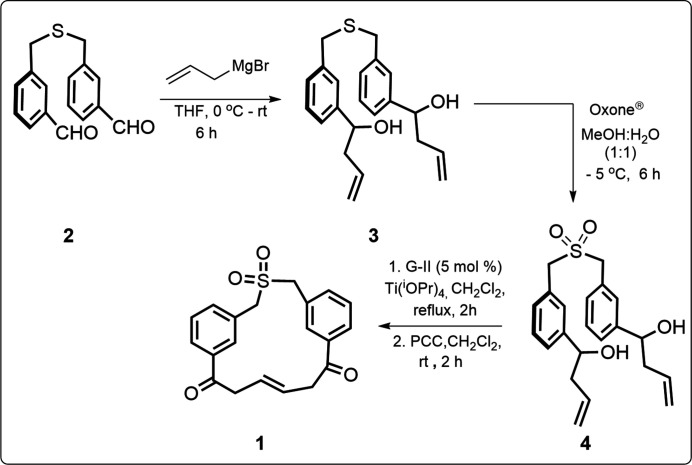
Synthesis of thia­meta­cyclo­phane **1**.

For the synthesis of the compound **1**, we started with the preparation of the di­aldehyde **2** (Kotha *et al.*, 2020 ▸). Later on, a Grignard reaction with **2** gave **3**, which on further oxidation provided the sulfone **4**. In an oven-dried, two-neck round-bottom flask, compound **4** (1eq., 50 mg) was dissolved in dry di­chloro­methane (20 ml). 1 Drop (0.1 eq.) of Ti(O^*i*^Pr)_4_ was added under an inert atmosphere and the reaction mixture was degassed by nitro­gen gas. After degasification, Grubbs’ second generation catalyst (5–10 mmol-%) was added, and the reaction mixture was refluxed. After completion of the reaction (TLC monitoring), the crude product was then subjected to oxidation by using pyridinium chloro­chromate (2.5 eq.) at room temperature. After completion of the reaction (TLC monitoring), the product was concentrated and purified by silica gel column chromatography using petroleum ether and ethyl acetate as the eluent to afford the desired compound **1** (Fig. 3 ▸). The single crystals were obtained by recrystallization in ethyl acetate and petroleum ether (1:2).

Yield 27 mg, 58%, m.p. 186–188°C, appearance: colourless solid, *R*_f_ = 0.5 (50% EtOAc–petroleum ether), ^1^H NMR 400 MHz, CDCl_3_) δ 8.04 (*d*, *J* = 8.0 Hz, 2H), 7.91 (*d*, *J* = 8.0 Hz, 2H), 7.56–7.51 (*m*, 4H), 5.83 (*t*, *J* = 3.2 Hz, 2H), 4.08 (*s*, 4H), 3.59 (*m*, 4H) p.p.m., ^13^C NMR (100 MHz, CDCl_3_) δ 197.1, 136.9, 135.4, 131.2, 130.1, 129.4, 128.7, 127.8, 57.1, 43.7 p.p.m., HRMS (ESI) *m*/*z* calculated C_20_H_18_O_4_SK[*M* + K]^+^ 393.0557, found 393.0551.

## Refinement

Crystal data, data collection and structure refinement details are summarized in Table 1 ▸.

## Supplementary Material

Crystal structure: contains datablock(s) I. DOI: 10.1107/S2414314620014649/bt4102sup1.cif

Structure factors: contains datablock(s) I. DOI: 10.1107/S2414314620014649/bt4102Isup2.hkl

CCDC reference: 2040913

Additional supporting information:  crystallographic information; 3D view; checkCIF report

## Figures and Tables

**Table 1:** Experimental details

Crystal data	
Chemical formula	C_20_H_18_O_4_S
*M*_r_	354.40
Crystal system, space group	Monoclinic, *P*2_1_/*n*
Temperature (K)	150
*a*, *b*, *c* (Å)	8.4257 (11), 9.0522 (9), 22.237 (2)
β (°)	98.303 (12)
*V* (Å^3^)	1678.3 (3)
*Z*	4
Radiation type	Mo *K*α
μ (mm^−1^)	0.22
Crystal size (mm)	0.15 × 0.09 × 0.03

Data collection	
Diffractometer	Rigaku Saturn724+
Absorption correction	Multi-scan (*CrysAlis PRO*; Rigaku, 2018 ▸)
*T*_min_, *T*_max_	0.921, 1.000
No. of measured, independent and observed [*I* > 2σ(*I*)] reflections	6670, 2942, 1671
*R*_int_	0.083
(sin θ/λ)_max_ (Å^−1^)	0.595

Refinement	
*R*[*F* ^2^ > 2σ(*F* ^2^)], *wR*(*F* ^2^), *S*	0.069, 0.165, 1.04
No. of reflections	2942
No. of parameters	226
H-atom treatment	H-atom parameters constrained
Δρ_max_, Δρ_min_ (e Å^−3^)	0.42, −0.42

Computer programs: *CrysAlis PRO* (Rigaku, 2018 ▸), *SHELXT* (Sheldrick, 2015*a* ▸), *SHELXL* (Sheldrick, 2015*b* ▸) and *OLEX2* (Dolomanov *et al.*, 2009 ▸).

## full crystallographic data

### Crystal data

**Table:**

C_20_H_18_O_4_S	*F*(000) = 744
*M**_r_* = 354.40	*D*_x_ = 1.403 Mg m^−^^3^
Monoclinic, *P*2_1_/*n*	Mo *K*α radiation, λ = 0.71073 Å
*a* = 8.4257 (11) Å	Cell parameters from 1833 reflections
*b* = 9.0522 (9) Å	θ = 2.4–26.4°
*c* = 22.237 (2) Å	µ = 0.22 mm^−^^1^
β = 98.303 (12)°	*T* = 150 K
*V* = 1678.3 (3) Å^3^	Block, colourless
*Z* = 4	0.14 × 0.09 × 0.03 mm

### Data collection

**Table:**

Rigaku Saturn724+ diffractometer	2942 independent reflections
Radiation source: fine-focus sealed X-ray tube, Enhance (Mo) X-ray Source	1671 reflections with *I* > 2σ(*I*)
Graphite monochromator	*R*_int_ = 0.083
Detector resolution: 28.5714 pixels mm^-1^	θ_max_ = 25.0°, θ_min_ = 2.4°
ω scans	*h* = −9→10
Absorption correction: multi-scan (CrysAlisPro; Rigaku, 2018)	*k* = −7→10
*T*_min_ = 0.921, *T*_max_ = 1.000	*l* = −18→26
6670 measured reflections	

### Refinement

**Table:**

Refinement on *F*^2^	Primary atom site location: structure-invariant direct methods
Least-squares matrix: full	Hydrogen site location: inferred from neighbouring sites
*R*[*F*^2^ > 2σ(*F*^2^)] = 0.069	H-atom parameters constrained
*wR*(*F*^2^) = 0.165	*w* = 1/[σ^2^(*F*_o_^2^) + (0.0393*P*)^2^ + 0.7221*P*] where *P* = (*F*_o_^2^ + 2*F*_c_^2^)/3
*S* = 1.04	(Δ/σ)_max_ < 0.001
2942 reflections	Δρ_max_ = 0.42 e Å^−^^3^
226 parameters	Δρ_min_ = −0.42 e Å^−^^3^
0 restraints	

### Special details

**Table:**

Geometry. All esds (except the esd in the dihedral angle between two l.s. planes) are estimated using the full covariance matrix. The cell esds are taken into account individually in the estimation of esds in distances, angles and torsion angles; correlations between esds in cell parameters are only used when they are defined by crystal symmetry. An approximate (isotropic) treatment of cell esds is used for estimating esds involving l.s. planes.
Refinement. H atoms were refined using a riding model with U(H)=1.2UeqC and with Caromatic—H = 0.95?Å or Cmethylene—H = 0.99?Å.

### Fractional atomic coordinates and isotropic or equivalent isotropic displacement parameters (Å^2^)

**Table:**

	*x*	*y*	*z*	*U*_iso_*/*U*_eq_	
S1	0.78574 (15)	0.72123 (11)	0.70216 (5)	0.0316 (4)	
O2	0.7211 (4)	0.8626 (3)	0.68124 (14)	0.0421 (9)	
O1	0.8964 (4)	0.7170 (3)	0.75787 (13)	0.0392 (9)	
O3	0.1830 (4)	0.3005 (3)	0.50373 (14)	0.0475 (10)	
O4	1.0566 (4)	−0.0096 (3)	0.62633 (17)	0.0515 (10)	
C6	1.0134 (5)	0.2434 (4)	0.64281 (18)	0.0267 (10)	
C14	0.3355 (6)	0.4516 (4)	0.57729 (19)	0.0305 (11)	
C1	0.8785 (5)	0.6454 (4)	0.64184 (18)	0.0267 (11)	
H1A	0.7948	0.6265	0.6067	0.032*	
H1B	0.9536	0.7194	0.6291	0.032*	
C8	0.9719 (6)	0.0980 (4)	0.6111 (2)	0.0325 (11)	
C7	0.9243 (5)	0.3719 (4)	0.62869 (18)	0.0249 (10)	
H7	0.8320	0.3692	0.5986	0.030*	
C15	0.2412 (5)	0.5744 (5)	0.5605 (2)	0.0323 (11)	
H15	0.1555	0.5682	0.5278	0.039*	
C2	0.9693 (5)	0.5035 (4)	0.65816 (18)	0.0250 (10)	
C19	0.4621 (5)	0.4625 (4)	0.62534 (18)	0.0267 (10)	
H19	0.5263	0.3782	0.6372	0.032*	
C4	1.1943 (5)	0.3788 (5)	0.7164 (2)	0.0347 (12)	
H4	1.2863	0.3821	0.7466	0.042*	
C13	0.2932 (6)	0.3062 (5)	0.5460 (2)	0.0352 (12)	
C3	1.1054 (5)	0.5051 (5)	0.7015 (2)	0.0315 (11)	
H3	1.1382	0.5952	0.7215	0.038*	
C9	0.8348 (5)	0.0863 (5)	0.5607 (2)	0.0357 (12)	
H9A	0.8362	−0.0127	0.5419	0.043*	
H9B	0.8486	0.1605	0.5291	0.043*	
C11	0.5455 (5)	0.1580 (4)	0.5472 (2)	0.0329 (11)	
H11	0.5545	0.1882	0.5069	0.040*	
C5	1.1485 (5)	0.2470 (4)	0.68701 (18)	0.0302 (11)	
H5	1.2091	0.1596	0.6970	0.036*	
C17	0.3971 (6)	0.7161 (4)	0.63881 (19)	0.0319 (11)	
H17	0.4166	0.8070	0.6600	0.038*	
C16	0.2725 (5)	0.7065 (5)	0.59171 (19)	0.0337 (12)	
H16	0.2076	0.7906	0.5804	0.040*	
C12	0.3844 (6)	0.1679 (4)	0.5677 (2)	0.0349 (12)	
H12A	0.3985	0.1652	0.6127	0.042*	
H12B	0.3200	0.0805	0.5525	0.042*	
C18	0.4949 (5)	0.5959 (4)	0.65603 (19)	0.0271 (10)	
C10	0.6757 (6)	0.1099 (4)	0.5816 (2)	0.0339 (12)	
H10	0.6680	0.0885	0.6229	0.041*	
C20	0.6247 (5)	0.6022 (4)	0.71042 (19)	0.0293 (11)	
H20A	0.6675	0.5013	0.7188	0.035*	
H20B	0.5760	0.6343	0.7462	0.035*	

### Atomic displacement parameters (Å^2^)

**Table:**

	*U*^11^	*U*^22^	*U*^33^	*U*^12^	*U*^13^	*U*^23^
S1	0.0419 (8)	0.0204 (6)	0.0310 (6)	−0.0001 (5)	0.0005 (6)	−0.0055 (5)
O2	0.054 (2)	0.0163 (14)	0.053 (2)	0.0032 (15)	−0.0005 (18)	−0.0051 (14)
O1	0.045 (2)	0.0354 (17)	0.0338 (18)	0.0011 (15)	−0.0077 (16)	−0.0115 (14)
O3	0.046 (2)	0.053 (2)	0.038 (2)	0.0074 (17)	−0.0131 (18)	−0.0068 (16)
O4	0.038 (2)	0.0275 (17)	0.085 (3)	0.0069 (16)	−0.002 (2)	−0.0016 (17)
C6	0.030 (3)	0.026 (2)	0.025 (2)	0.000 (2)	0.006 (2)	0.0027 (18)
C14	0.033 (3)	0.030 (2)	0.028 (3)	0.002 (2)	0.003 (2)	0.0018 (19)
C1	0.037 (3)	0.019 (2)	0.023 (2)	−0.003 (2)	0.002 (2)	−0.0017 (18)
C8	0.037 (3)	0.026 (2)	0.035 (3)	−0.001 (2)	0.008 (2)	−0.003 (2)
C7	0.024 (3)	0.027 (2)	0.023 (2)	0.001 (2)	0.000 (2)	0.0004 (18)
C15	0.030 (3)	0.041 (3)	0.027 (3)	0.007 (2)	0.005 (2)	0.009 (2)
C2	0.032 (3)	0.023 (2)	0.021 (2)	−0.003 (2)	0.005 (2)	0.0038 (18)
C19	0.027 (3)	0.024 (2)	0.030 (2)	0.007 (2)	0.009 (2)	0.0038 (18)
C4	0.026 (3)	0.046 (3)	0.030 (3)	−0.005 (2)	−0.001 (2)	−0.001 (2)
C13	0.036 (3)	0.044 (3)	0.026 (2)	0.006 (2)	0.003 (2)	−0.001 (2)
C3	0.032 (3)	0.028 (2)	0.034 (3)	0.000 (2)	0.004 (2)	−0.0016 (19)
C9	0.037 (3)	0.029 (2)	0.040 (3)	0.006 (2)	0.001 (2)	−0.007 (2)
C11	0.037 (3)	0.025 (2)	0.036 (3)	0.000 (2)	0.001 (2)	−0.008 (2)
C5	0.030 (3)	0.031 (2)	0.030 (2)	−0.002 (2)	0.004 (2)	0.005 (2)
C17	0.038 (3)	0.027 (2)	0.032 (3)	0.009 (2)	0.010 (2)	0.003 (2)
C16	0.036 (3)	0.031 (2)	0.034 (3)	0.012 (2)	0.004 (2)	0.014 (2)
C12	0.043 (3)	0.029 (2)	0.032 (3)	−0.005 (2)	0.001 (2)	−0.003 (2)
C18	0.028 (3)	0.027 (2)	0.028 (2)	−0.001 (2)	0.009 (2)	0.0023 (19)
C10	0.039 (3)	0.027 (2)	0.031 (3)	0.002 (2)	−0.006 (2)	−0.0037 (19)
C20	0.038 (3)	0.025 (2)	0.026 (2)	0.001 (2)	0.007 (2)	0.0033 (18)

### Geometric parameters (Å, º)

**Table:**

S1—O2	1.441 (3)	C4—H4	0.9500
S1—O1	1.439 (3)	C4—C3	1.380 (6)
S1—C1	1.785 (4)	C4—C5	1.388 (5)
S1—C20	1.763 (4)	C13—C12	1.511 (6)
O3—C13	1.223 (5)	C3—H3	0.9500
O4—C8	1.226 (5)	C9—H9A	0.9900
C6—C8	1.510 (5)	C9—H9B	0.9900
C6—C7	1.395 (5)	C9—C10	1.497 (6)
C6—C5	1.393 (5)	C11—H11	0.9500
C14—C15	1.386 (6)	C11—C12	1.496 (6)
C14—C19	1.400 (5)	C11—C10	1.318 (5)
C14—C13	1.507 (6)	C5—H5	0.9500
C1—H1A	0.9900	C17—H17	0.9500
C1—H1B	0.9900	C17—C16	1.375 (6)
C1—C2	1.512 (5)	C17—C18	1.385 (5)
C8—C9	1.492 (6)	C16—H16	0.9500
C7—H7	0.9500	C12—H12A	0.9900
C7—C2	1.385 (5)	C12—H12B	0.9900
C15—H15	0.9500	C18—C20	1.510 (6)
C15—C16	1.389 (6)	C10—H10	0.9500
C2—C3	1.388 (6)	C20—H20A	0.9900
C19—H19	0.9500	C20—H20B	0.9900
C19—C18	1.395 (5)		
			
O2—S1—C1	106.56 (19)	C2—C3—H3	119.2
O2—S1—C20	108.4 (2)	C4—C3—C2	121.5 (4)
O1—S1—O2	117.98 (18)	C4—C3—H3	119.2
O1—S1—C1	109.7 (2)	C8—C9—H9A	109.0
O1—S1—C20	107.84 (19)	C8—C9—H9B	109.0
C20—S1—C1	105.7 (2)	C8—C9—C10	112.8 (4)
C7—C6—C8	122.7 (4)	H9A—C9—H9B	107.8
C5—C6—C8	117.4 (4)	C10—C9—H9A	109.0
C5—C6—C7	119.8 (4)	C10—C9—H9B	109.0
C15—C14—C19	119.5 (4)	C12—C11—H11	118.0
C15—C14—C13	119.2 (4)	C10—C11—H11	118.0
C19—C14—C13	121.2 (4)	C10—C11—C12	123.9 (4)
S1—C1—H1A	108.7	C6—C5—H5	120.2
S1—C1—H1B	108.7	C4—C5—C6	119.7 (4)
H1A—C1—H1B	107.6	C4—C5—H5	120.2
C2—C1—S1	114.1 (3)	C16—C17—H17	119.4
C2—C1—H1A	108.7	C16—C17—C18	121.3 (4)
C2—C1—H1B	108.7	C18—C17—H17	119.4
O4—C8—C6	118.5 (4)	C15—C16—H16	119.9
O4—C8—C9	120.5 (4)	C17—C16—C15	120.3 (4)
C9—C8—C6	121.0 (4)	C17—C16—H16	119.9
C6—C7—H7	119.7	C13—C12—H12A	108.9
C2—C7—C6	120.6 (4)	C13—C12—H12B	108.9
C2—C7—H7	119.7	C11—C12—C13	113.2 (4)
C14—C15—H15	120.1	C11—C12—H12A	108.9
C14—C15—C16	119.8 (4)	C11—C12—H12B	108.9
C16—C15—H15	120.1	H12A—C12—H12B	107.8
C7—C2—C1	121.5 (4)	C19—C18—C20	119.6 (4)
C7—C2—C3	118.7 (4)	C17—C18—C19	118.5 (4)
C3—C2—C1	119.8 (3)	C17—C18—C20	121.7 (4)
C14—C19—H19	119.7	C9—C10—H10	117.5
C18—C19—C14	120.6 (4)	C11—C10—C9	125.0 (4)
C18—C19—H19	119.7	C11—C10—H10	117.5
C3—C4—H4	120.2	S1—C20—H20A	108.6
C3—C4—C5	119.7 (4)	S1—C20—H20B	108.6
C5—C4—H4	120.2	C18—C20—S1	114.8 (3)
O3—C13—C14	119.6 (4)	C18—C20—H20A	108.6
O3—C13—C12	120.3 (4)	C18—C20—H20B	108.6
C14—C13—C12	120.0 (4)	H20A—C20—H20B	107.6
			
S1—C1—C2—C7	117.0 (4)	C7—C2—C3—C4	−1.1 (7)
S1—C1—C2—C3	−66.1 (5)	C15—C14—C19—C18	−0.6 (7)
O2—S1—C1—C2	173.6 (3)	C15—C14—C13—O3	4.7 (7)
O2—S1—C20—C18	50.0 (3)	C15—C14—C13—C12	−174.0 (4)
O1—S1—C1—C2	44.9 (4)	C19—C14—C15—C16	−0.4 (7)
O1—S1—C20—C18	178.8 (3)	C19—C14—C13—O3	−179.2 (4)
O3—C13—C12—C11	103.5 (5)	C19—C14—C13—C12	2.1 (7)
O4—C8—C9—C10	115.5 (5)	C19—C18—C20—S1	118.1 (4)
C6—C8—C9—C10	−66.8 (5)	C13—C14—C15—C16	175.8 (4)
C6—C7—C2—C1	177.5 (4)	C13—C14—C19—C18	−176.7 (4)
C6—C7—C2—C3	0.6 (6)	C3—C4—C5—C6	0.0 (7)
C14—C15—C16—C17	0.4 (7)	C5—C6—C8—O4	1.3 (6)
C14—C19—C18—C17	1.5 (6)	C5—C6—C8—C9	−176.4 (4)
C14—C19—C18—C20	175.8 (4)	C5—C6—C7—C2	0.2 (6)
C14—C13—C12—C11	−77.8 (5)	C5—C4—C3—C2	0.8 (7)
C1—S1—C20—C18	−63.9 (3)	C17—C18—C20—S1	−67.7 (5)
C1—C2—C3—C4	−178.1 (4)	C16—C17—C18—C19	−1.5 (7)
C8—C6—C7—C2	−178.5 (4)	C16—C17—C18—C20	−175.7 (4)
C8—C6—C5—C4	178.3 (4)	C12—C11—C10—C9	174.3 (4)
C8—C9—C10—C11	154.2 (4)	C18—C17—C16—C15	0.5 (7)
C7—C6—C8—O4	−180.0 (4)	C10—C11—C12—C13	139.7 (4)
C7—C6—C8—C9	2.3 (7)	C20—S1—C1—C2	−71.2 (3)
C7—C6—C5—C4	−0.5 (6)		
